# Meeting abstracts from botanical symposium 2022: exploring innovative approaches in Chinese medicines—from bench to bedside

**DOI:** 10.1186/s13020-023-00796-8

**Published:** 2023-09-01

## P1 Acteoside from kidney-tonifying Chinese herbal medicine *Cistanches Herba* promotes myogenic differentiation in C2C12 myoblasts

### Chun Au-Yeung^1^, Christina Chui-Wa Poon^1,2^, Man-Sau Wong^1,2^

#### ^1^Department of Applied Biology and Chemical Technology, The Hong Kong Polytechnic University, Hung Hom, Kowloon, Hong Kong SAR, PRC; ^2^Research Centre for Chinese Medicine Innovation, The Hong Kong Polytechnic University, Hung Hom, Kowloon, Hong Kong SAR, PRC

##### **Correspondence:** Christina Chui-Wa Poon (christina.poon@polyu.edu.hk); Man-Sau Wong (man-sau.wong@polyu.edu.hk)

*Chinese Medicine* 2023, **18(Suppl 1):**P1

Sarcopenia is a progressive muscle condition characterised by the degenerative decline in skeletal muscle mass and function which arises from immobility and ageing. It has become a global health crisis with the growing older population. *Cistanches Herba* (Rou Cong-Rong), holoparasitic plant of *Cistanche deserticola*, is classified as a kidney-tonifying agent in traditional Chinese medicine and is commonly used to treat infertility and muscle weakness. It has various pharmacological activities, such as anti-osteoporotic, antioxidative and neuroprotective effects. Acteoside is one of the bioactive compounds found in *Cistanches Herba* that may account for its protective effects in skeletal muscle. The aim of this study is to determine the myogenic effect of acteoside in C2C12 myoblasts. Acteoside at 500 nM could promote myogenic differentiation significantly in differentiating C2C12 myoblasts by increasing the number and area of myotubes by 17% and 18% respectively (*p* < 0.05). These results provide evidence for supporting the use of acteoside for and the management of sarcopenia. Further study will be needed to determine the in vivo effects of acteoside and *Cistanches Herba* in sarcopenic animal model and to evaluate the underlying mechanisms of their protective effects in skeletal muscle.

## P2 An investigation of the pharmacological effects of Danggui Buxue Tang on bEnd.3 cells under oxygen–glucose deprivation and reoxygenation (OGD/R)

### Karry Yuen-Ching Cheung^1,2^, Hei-Yin Wong^1,2^, Bun Tsoi^1,2^, Sai-Wang Seto^1,2,3^

#### ^1^Department of Applied Biology and Chemical Technology, The Hong Kong Polytechnic University, Hong Kong SAR, China; ^2^Research Centre for Chinese Medicine Innovation, The Hong Kong Polytechnic University, Hong Kong SAR, China; ^3^NICM Health Research Institute, Western Sydney University, Penrith, NSW, 2751, Australia

##### **Correspondence:** Sai-Wang Seto (saiwang.seto@polyu.edu.hk)

*Chinese Medicine* 2023, **18(Suppl 1):**P2

Menopause is the permanent cessation of menses that is characterized by the decline of ovarian follicular activity and hormone fluctuation. The significant reduction in oestrogen is often considered as the major risk factor for vascular dementia (VaD) in menopausal women. VaD is characterized by cerebral hypoperfusion and disruption of the blood–brain barrier (BBB). Currently, hormone replacement therapy (HRT) is the only treatment to alleviate distressing menopausal symptoms. However, large body of evidence showed that chronic HRT could increase the risk of various chronic diseases (eg. dementia and hypertension), limiting its clinical use in menopause symptoms management. Hence, alternative therapy that is safe and effective is urgently needed. Danggui Buxue Tang (DBT) is an ancient Chinese herbal decoction consisting of 2 herbs, *Astragali Radix* (Huangqi) and *Angelicae Sinensis Radix* (Danggui). DBT has been shown to possess estrogenic and osteogenic effects and thus a popular herbal formula among menopausal women in China and other Asian countries.

However, its pharmacological effects on cerebral endothelial cells have not been fully elucidated. We hypothesized that DBT could increase proliferation, migration and enhance tight junction molecules expression in OGD/R-insulted mouse brain endothelial (bEnd.3) cells.

The effects of DBT on bEnd.3 cell proliferation and migration were assessed by MTT and wound scratch assay, respectively. Protein expression of tight junction proteins (ZO-1 and occludin) were examined using western blotting. Our result showed that DBT (0.01–10 mg/ml) increased proliferation and migration of bEnd.3 cells in a concentration-dependent manner. OGD/R markedly reduced cell viability by 60%. DBT (0.01–10 mg/ml) suppressed the OGD/R-induced cell death in a concentration-dependent manner. Moreover, OGD/R reduced ZO-1 protein expression, which was normalised by DBT (0.1–3 mg/ml). In conclusion, our results suggest that DBT could be a potential therapeutic strategy for vascular dementia via angiogenesis and protection of BBB integrity.

## P3 Investigation of the anti-microbial properties with modulating host macrophages phagocytosis of *Lantana camara* L. water extracts

### Patrick Pak-Ting Hau^1,2^, Carsten Tsun-Ka Kwok^3^, Ray Chun-Wai Yu^2^, Fiona Wong^3^, Franklin Wang-Ngai Chow^1,2^, Sai-Wang Seto^1,3^

#### ^1^Research Centre for Chinese Medicine Innovation, The Hong Kong Polytechnic University, Hong Kong SAR, China; ^2^Department Health Technology and Informatics, The Hong Kong Polytechnic University, Hong Kong SAR, China; ^3^Department of Applied Biology and Chemical Technology, The Hong Kong Polytechnic University, Hong Kong SAR, China

##### **Correspondence:** Franklin Wang-Ngai Chow (franklin.chow@polyu.edu.hk); Sai-Wang Seto (saiwang.seto@polyu.edu.hk)

*Chinese Medicine* 2023, **18(Suppl 1):**P3

**Background:** *Lantana camara* L. is effective in inhibiting microbes by the highly-toxic flavonoids in the ethanol extract of its leaves, however there is limited study on investigating the effect of *L. camara* L. water extract from leaves (LWB) on host–pathogen interactions in macrophages. This study aims to examine effect of LWB on modulating host–pathogen interactions in macrophages.

**Methods:** First, the cytotoxicity of LWB in macrophages was examined by MTT-assay. Second, the minimal-inhibitory-concentration (MIC) of LWB against *Staphylococcus aureus* (SA) or *Candida krusei* (CK) was assayed by broth microdilutions. Third, the phagocytotic rates of macrophages against SA or CK were determined by establishing a co-culture model with 2-h incubation, followed by measurement of colony-forming-units (CFU). Finally, cell supernatant was collected and used to measure nitric-oxide (NO) levels by Griess reagent.

**Results and conclusion:** LWB was non cytotoxic up to 2 mg/ml. The MIC of LWB to inhibit SA was 5 mg/ml while LWB was unable to inhibit CK even in 10 mg/ml. However, 2 mg/ml LWB markedly reduced CFU of SA or CK by 5.8 (p < 0.05) and 48.9 (p < 0.001) folds in the treated group respectively, when compared to the control group. In addition, LWB significantly stimulated NO production of macrophages approximately 219 (p < 0.0001) and 40-folds (p < 0.01) in the SA or CK infected-group respectively when compared with the infected-group without LWB treatment. In summary, LWB is a potential non-toxic TCM in assisting macrophages to eliminate microbes that may be a useful TCM herbs for management microbial infections.

## P4 Epimedium aqueous extract disrupted osteoclast differentiation, maturation and function via inhibition of autophagy

### Xueling Hu^1,2^, Wenqiang Zhong^1,2^, Bingjie Luo^1,2^, Weiwen Lin^1,2^, Ziling Tang^3^, Yangyang Zhang^3^, Jie Zhang^3^, Zuocheng Qiu^3^, Li Yang^1,2^

#### ^1^College of Pharmacy, Jinan University, Guangdong, China; ^2^Guangdong Provincial Key Laboratory of Traditional Chinese Medicine Informatization, Jinan University, Guangzhou, China; ^3^Guangzhou Key Laboratory of Formula-Pattern of Traditional Chinese Medicine, School of Traditional Chinese Medicine, Jinan University, Guangzhou, China

##### **Correspondence:** Zuocheng Qiu (zcqiu@jnu.edu.cn); Li Yang (doctormokey@126.com)

*Chinese Medicine* 2023, **18(Suppl 1):**P4

Epimedium is a classic kidney tonic traditional Chinese medicine used as an antiosteoporosis agent in China. The main ingredients include icariin, pilgrimage, bhoside and so on. Autophagy plays an important roles in the process of osteoclast differentiation and resorption, and studies have shown that inhibition of autophagy can lead to OC dysfunction and reduce OC bone resorption.

**Objectives:** Based on the theory of “kidney governs bone marrow”, this study aims to explore the effects of Epimedium on osteoclastic differentiation and determine if its underlying mechanism is related to autophagy in vitro.

**Methods:** Epimedium aqueous extract was obtained by reflux extraction, lyophilized to obtain lyophilized powder, and the components of lyophilized powder were detected by comparing with standards in using **HPLC**; then three-month-old SD rats was used to operated, ovariectomized model. After gavage for 12 weeks, the femur sample was used to perform **immunohistochemical** analysis; RAW264.7 is administered after 12 h of plating as the density of 30,000 cells/mL. **CCK-8 staining** was measured after 2 days of HEF administration, and **TRAP staining** was measured after osteoclastic induction and HEF administration for 4 days; 6-well plate was plated at the plating density of 40,000 cells/mL and administration after 12 h, drug changed every two days, mRNA and proteins extracted on the third day to measure osteoclast-related and autophagy-related gene and proteins expression by **qPCR** and **WB**.

**Result:** Extraction, lyophilization and qualitative detection of epimedium aqueous extract through HPLC, it can be guided that the main ingredients of epimedium are retained in the freeze–dried powder.

Epimedium aqueous extraction group can significantly reduce the CTSK activity of OVX rats compared with the model group.

Through cck8 and TRAP staining, it was found that lyophilized powder of Epimedium showed a dose-dependent effect on cell proliferation and osteoclastic differentiation of RAW264.7 after a certain dose. Finally, the dosing concentration of 100 μg/mL was determined as a subsequent mechanistic study.

The results showed that HEF could significantly reduce the expression of osteoclast-related mRNA and proteins and negatively regulate autophagy.

**Conclusions:** This experiment proves that the kidney tonic Chinese medicine Epimedium can inhibit the osteoclastic differentiation via regulating autophagy related genes and proteins, which partly reveals the anti-resorption mechanism of epimedium on bone.

## P5 The phytoestrogenic potential of *Selaginella moellendorffii* and its mechanism

### Shijun Yuan, Zihan Li, Keli Chen, Juan Li

#### Hubei Province Key Laboratory of Traditional Chinese Medicine Resource and Chemistry; Department of Pharmacy, Hubei University of Chinese Medicine, Wuhan, Hubei, China

##### **Correspondence:** Juan Li (lz198207@126.com)

*Chinese Medicine* 2023, **18(Suppl 1):**P5

Phytoestrogens, mainly classified as flavonoids, lignans, coumarins, and stilbenes, are structurally and functionally similar to endogenous estrogens. *Selaginella moellendorffii* Hieron, rich in flavonoids, has been used to treat osteoporosis, idiopathic thrombocytopenic purpura, and chronic inflammation. This study aims to investigate estrogen-like constituents of *Selaginella moellendorffii* and the possible mechanism. HPLC and UPLC-MS analysis were applied to identify the main components. The estrogenic effects were examined using a recombinant yeast screening assay and an E-screen cell proliferation assay. We identified amentoflavone, robusta biflavone-4′-methyl ether, 5 apigenin glycosides in HPLC chromatograms, and 18 compounds (9 flavonoids, 6 phenylpropanoids, 2 alkaloids, and 1 nucleoside) in UPLC-MS analysis. The aqueous and n-butanol extract showed estrogen agonistic activity in the yeast screening and E-SCREEN assay. In contrast, ethanol and ethyl acetate extract have no significant estrogen activity. The aqueous extract, n-butanol extract, apigenin, and apigenin flavonoids induced cell proliferation in ER-positive MCF-7 and T-47-D cells but not in ER-negative SK-BR-3 and MDA-MB-231 cells. Meanwhile, the n-butanol extract could improve the protein expression of ERα, PI3K, p-AKT, and p-mTOR. However, these effects could be blocked by ER antagonist or ERα antagonist but not be blocked by ERβ antagonist. In conclusion, the apigenin flavonoids might be the critical estrogenic constituents, and the ERα induction via the PI3K/Akt/mTOR signaling pathways may be a potential mechanism underlying the estrogen-like effects.

**Acknowledgements**

This work was funded by the National Natural Science Foundation of China (81903921).

## P6 Cascade enzymatic synthesis of rare biflavonoid glycosides with improved anti-tumor effects

### Wei Huang^1,2^, Su Xu^1^, Xiran Xiong^1^, Juan Li^1^

#### ^1^Hubei Province Key Laboratory of Traditional Chinese Medicine Resource and Chemistry; Department of Pharmacy, Hubei University of Chinese Medicine, Wuhan, Hubei, China; ^2^Ministry of Education Key Laboratory of Combinatorial Biosynthesis and Drug Discovery, School of Pharmaceutical Sciences, Wuhan University, Wuhan, China

##### **Correspondence:** Juan Li (lz198207@126.com)

*Chinese Medicine* 2023, **18(Suppl 1):**P6

Biflavonoids belong to a subclass of the plant flavonoids family and possess various critical biological properties. Although biflavonoids significantly impact human health, lower absorption and poor water solubility have hampered their applications considerably. Glycosylation usually improves pharmacodynamic and pharmacokinetic properties; however, biflavonoid glycosides are rare in plants. In this study, we established an efficient enzymatic cascade for biflavonoid production consisting of *O*-glycosyltransferase UGT74AN2 from *Calotropis gigantea* and sucrose synthase *At*SuSy from *Arabidopsis thaliana*^1^. The highest conversion rate (> 85%) of bifavonoid glycosides was archived at an optimal reaction time 2 h, temperature 35 °C, and a 3:1 sucrose/UDP molar ratio. Six biflavonoids glycosides were isolated and structurally identified by MS and NMR. The water solubilities of AF-1, HAF-1, and KF-1 were 24.6, 30.4, and 27.3 times higher than corresponding aglycones, respectively. Moreover, AF-1 and HAF-1 showed higher anti-tumor effects than corresponding aglycones in prostate cells. This study not only established an efficient enzymatic approach for biflavonoids glycoside production but also provided a series of potential small molecules for drug discovery.

**Reference**

Huang W, He Y, Jiang R, et al. Functional and Structural Dissection of a Plant Steroid 3-O-Glycosyltransferase Facilitated the Engineering Enhancement of Sugar Donor Promiscuity. *ACS Catalysis*, **2022**, *12*(5): 2927–2937.

**Acknowledgements**

This work was funded by the National Natural Science Foundation of China (81903921).

## P7 A preliminary pharmacophylogenetic study of medicinal plants from genus *Oxytropis* DC.

### Congying Huang^1^, Chunhong Zhang^1^, Aruhan^2,3^, D. Tsend-Ayush^3^, Minhui Li^1,4^, Peigen Xiao^5^

#### ^1^Department of Pharmacy, Baotou Medical College, Baotou, 014040, China; ^2^Baotou Mongolian and Chinese Medicine Hospital, Baotou, 014040, China; ^3^International School of Mongolian Medicine, MNUMS, Ulaanbaatar, 999097-15141, Mongolia; ^4^Inner Mongolia Autonomous Region Hospital of Traditional Chinese Medicine, Hohhot, 010020, China; ^5^Institute of Medicinal Plant Development, Chinese Academy of Medical Sciences and Peking Union Medical College, Beijing, 100193, China

##### **Correspondence:** Minhui Li (prof_liminhui@yeah.net); Peigen Xiao (pgxiao@implad.ac.cn)

*Chinese Medicine* 2023, **18(Suppl 1):**P7

*Oxytropis* DC., a genus of Leguminosae, is a perennial herb, semi shrub or dwarf shrub, which normally distributed in temperate regions of Europe, Asia and North America. There are more than 350 species in the world, some of which have significant pharmacological activities and are widely used in traditional medicine^[1]^. This study mainly discussed 19 medicinal species, and explored the relationship between the traditional efficacy, chemical constituents, pharmacological effects and genetic relationship. The bibliometrics method was used to collect the information of 19 medicinal species. The ITS sequence of nucleotides was downloaded to construct phylogenetic tree. Based on the innovative correlation analysis, the phylogenetic relationship of medicinal plants of *Oxytropis* DC*.* was preliminarily discussed^[2]^. According to genetic theory, 19 medicinal plants can be divided into three groups. The first group includes 16 species, including *Oxytropis falcata*, *Oxytropis microphylla* and *Oxytropis oxyphylla*. They contain flavonoids, alkaloids and volatile oils, and can play the role of clearing heat, detoxification, detumescence and analgesia. The second group includes *Oxytropis racemosa* and *Oxytropis tragacanthoides*, in which flavonoids, alkaloids can regulate gastrointestinal function. The third group is *Oxytropis coerulea*, in which saponins and flavonoids can strengthen the body through immune regulation and play a nourishing role. *Oxytropis* DC*.* is rich in medicinal plant resources. The genetic analysis of medicinal plants based on multi-perspective is of great significance to deeply understand this genus, identify the drug base source, and discover new drugs or alternative drugs^[3]^.

**Figure 1 (abstract P7):**
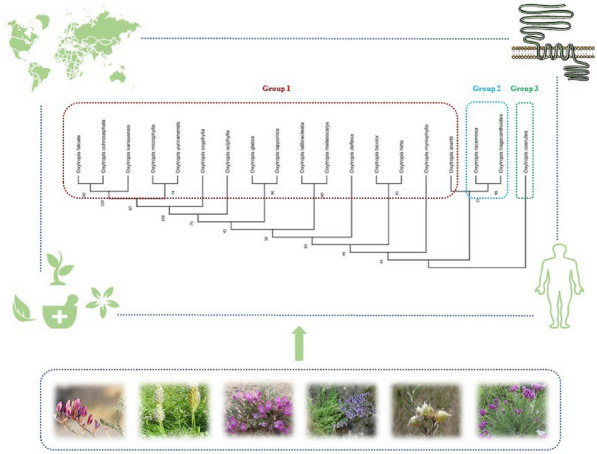
The pharmacophylogenetic research of medicinal plants from genus *Oxytropis* DC.

**References**

1. Liu, B. Medicinal Plant of Oxytropis in China and Its Modernized Study. *Chinese Wild Plant Resources,* **1997,** *02*, 17–20.
2. Xiao, P. G., Li, M. H., Hao, D.C., He, C. N., Xu, L. J. Theoretical Innovation and Application Practice of Pharmacophylogeny. *Modern Chinese Medicine, 2021, 23,* 1499–1505.
3. Gong, X., Yang, M., He, C. N., Bi, Y. Q., Zhang, C. H., Li, M. H., Xiao, P. G. Plant Pharmacophylogeny: Review and Future Directions. *Chinese Journal of Integrative Medicine, 2022, 6,* 567–574.

## P8 To explore the medicinal values of plants in hyoscyameae based on the pharmacophylogeny

### Siqi Li^1^, Chunhong Zhang^1^, Bin Qiu^2^, Aruhan^4^, D. Tsend-Ayush^4^, Minhui Li^1,5^, Peigen Xiao^3^

#### ^1^Department of Pharmacy, Baotou Medical College, Baotou, 014040, China; ^2^Yunnan University of Chinese Medicine, Kunming, 650500, China; ^3^Institute of Medicinal Plant Development, Chinese Academy of Medical Sciences and Peking Union Medical College, Beijing, 100193, China; ^4^International School of Mongolian Medicine, MNUMS, Ulaanbaatar, 999097-15141, Mongolia; ^5^Inner Mongolia Autonomous Region Hospital of Traditional Chinese Medicine, Hohhot, 010020, China

##### **Correspondence:** Minhui Li (prof_liminhui@yeah.net); Peigen Xiao (pgxiao@implad.ac.cn)

*Chinese Medicine* 2023, **18(Suppl 1):**P8

Solanaceae has about 96 genera and 3000 species, which are widely distributed in temperate and tropical regions of the world. Hyoscyameae are rich in atropine alkaloids and are an important medicinal plant group of Solanaceae ^[1]^ (Figure 1A). This paper summarized the traditional curative effect, chemical composition and pharmacological effect, and discussed the pharmacophylogeny of Hyoscyameae. Through the analysis of chemical components, it is found that each genus of the Hyoscyameae contains one or more atropine alkaloids. The main effect of this large group is spasmolysis and pain relief. In modern pharmacology, it is commonly used to treat various stomachaches ^[1]^. In addition, each genus is not exactly the same in chemical composition, traditional curative effect and pharmacological effect. As shown in Figure 1B, medicinal plants in group1 mainly contain scopolamine and atropine, which have obvious effects of dispelling wind and cold, and are mostly used for rheumatoid arthritis. The plants in group2 mainly contain hyoscyamine, which has the effect of calming nerves and asthma, and is commonly used for cough. According to the phylogenetic study on the plants of Hyoscyameae, these plants can be divided into three groups ^[2]^. In addition to medicinal plants, some plants of the Hyoscyameae have not yet reported their chemical constituents and pharmacological effects. According to the pharmacophylogeny, we can reasonably speculate on their constituents and pharmacological effects, and further develop the medicinal values of these plants ^[3]^. In addition, the collation of the plants of the Hyoscyameae containing atropine alkaloids can provide scientific basis for the further research and clinical application of this group.

**Figure 1 (abstract P8):**
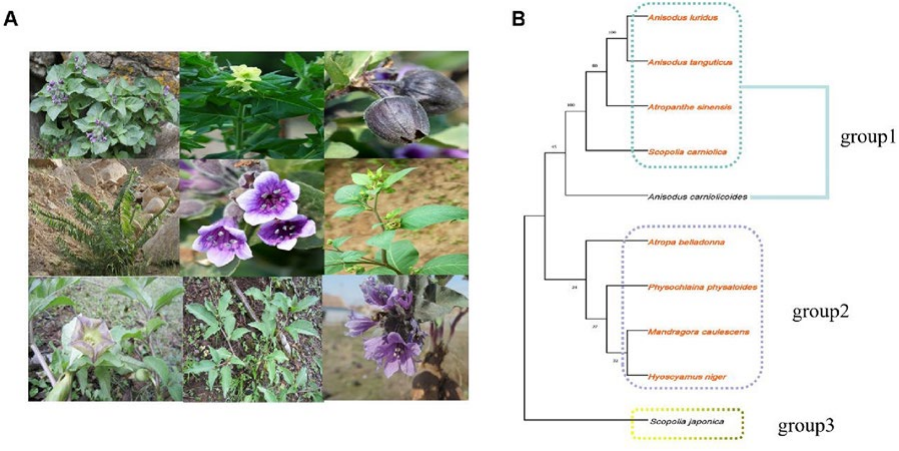
Phylogenetic tree and morphological characteristics of Hyoscyameae. **A** Morphological characteristics of plants of Hyoscyameae; **B** phylogenetic tree of the plants of Hyoscyameae, the plants highlighted in orange are medicinal plants, and the rest are non-medicinal plants

**References**

1. Zang, E.H.; Li, Q.Y.; Xu, J.F.; Zhang, Y.; Jiang, L.L.; Li, X.; Zhang, M.X.; Liu, Y.C.; Wu, Q.J.; Liu, Z.H.; Li, M.H.; Xiao, P.G. A Preliminary Pharmacophylogenetic Study of Solanaceae Medicinal Plants Containing Tropane Alkaloids. C*hina Journal of Chinese Materia Medica,* **2021**, *46*, 4344–4359.
2. Gong, X., Yang, M., He C.N., Bi, Y.Q., Zhang, C.H., Li, M.H., Xiao, P.G. Plant Pharmacophylogeny: Review and Future Directions. *Chinese Journal of Integrative Medicine*, **2022**, *6*, 567–574.
3. Xiao, P. G., Li, M. H., Hao, D.C., He, C. N., Xu, L. J. Theoretical Innovation and Application Practice of Pharmacophylogeny. *Modern Chinese Medicine*, **2021**, *23*, 1499–1505.

## P9 In vitro and in vivo evaluation of a traditional Chinese medicine formula Si Ben Cao for skin application

### Yu-Shan Lin, Hui-Hui Xiao, Guo-Qing Chen

#### State Key Laboratory of Chinese Medicine and Molecular Pharmacology (Incubation), Shenzhen Research Institute, The Hong Kong Polytechnic University, Hong Kong SAR, China

##### **Correspondence:** Hui-Hui Xiao (huihui.xiao@polyu.edu.hk); Guo-Qing Chen (guoqing.chen@polyu.edu.hk)

*Chinese Medicine* 2023, **18(Suppl 1):**P9

Recent years have seen an increase in the popularity of cosmetic products with skin whitening function derived from natural sources^1−2^. However, many products are not actually proven to be effective. In this study, Si Ben Cao (SBC), a traditional Chinese medicine formula composed of four herbs, was evaluated for its safety and potential benefits to skin application. Results showed that aqueous extract of SBC had no cytotoxicity even after 96 h administration, and it did not cause irritations and allergies to rats, both of which indicated that SBC extract is safe to apply in the skin. Moreover, SBC extract showed strong inhibitory effects on cellular tysosinase activity and the production of melanin. In addition, SBC extract was able to absorb Ultraviolet (UV) ray and have anti-oxidant properties. Collectedly, our study indicated that SBC extract is safe to apply in the skin for the purpose of skin whitening and health benefits.

**References**

1. Qian, W.; Liu, W.; Zhu, D.; Cao, Y.; Tang, A.; Gong, G.; Su, H., Natural skin-whitening compounds for the treatment of melanogenesis (Review). *Exp Ther Med* **2020,** *20* (1), 173–185.
2. Ko, C. Y.; Chao, J.; Chen, P. Y.; Su, S. Y.; Maeda, T.; Lin, C. Y.; Chiang, H. C.; Huang, S. S., Ethnobotanical Survey on Skin Whitening Prescriptions of Traditional Chinese Medicine in Taiwan. *Front Pharmacol* **2021,** *12*, 736370.

## P10 Study on insect resistant activity and mechanism of natural products based on visual analysis

### Xing Li^3^, Chunyan Guo^4^, Letai Yi^2^, Lijuan Lv^1^

#### ^1^Department of Basic Science, Tianjin Agricultural University, Tianjin, 300392, China; ^2^College of Mongolian medicine and pharmacy, Inner Mongolia Medical University, Hohhot, 010100, China; ^3^Department of Pharmacy, Baotou Medical College, Baotou, 014040, China; ^4^College of Pharmacy, Qiqihar Medical University, Qiqihar, 161000, China

##### **Correspondence:** Letai Yi (yiletai@126.com); Lijuan Lv (lv_lijuan@aliyun.com)

*Chinese Medicine* 2023, **18(Suppl 1):**P10

With the rapid growth of the global population, the challenge of ensuring food security has received widespread attention. Crop diseases and insect pests are among the primary reasons for the large-scale loss of agricultural production. A visual analysis makes it apparent that although chemical insecticides have a certain effect on controlling crop pests, common pesticides in the market, such as chlorphenamine and parathion, are toxic, and the target organisms readily develop resistance against these chemicals [1]. Chemical pesticides cannot fundamentally solve the food security problem; moreover, they can worsen the current scenario by polluting the environment and endangering human health [2]. Therefore, it is urgent to find green and efficient insecticides that can replace chemical pesticides. When it comes to environment-friendly pesticides, natural products to control pests have naturally become the first choice. These natural products mainly act on digestive system, respiratory system, and nervous system of the pests, thereby killing them. This paper makes a comprehensive and detailed analysis of the main areas, hot countries, organizational cooperation, citation sources, and cutting-edge trends involved in the study of pest resistance of natural products and skillfully combines data from the literature and examples based on visual analysis. In this paper, the anti-pest effects and mechanisms of natural products with anti-pest activities such as flavonoids, terpenoids and alkaloids were reviewed. This novel compilation of data and literature is adequate to offer a solid foundation and reliable ideas for researchers to study the application and development of natural products in pest control.
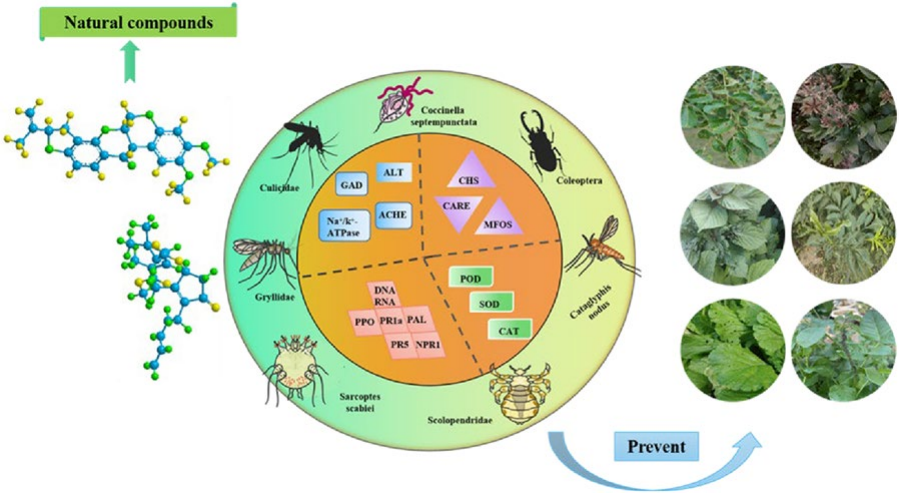


**References**

1. Kumar, S.; Bhanjana, G.; Sharma, A. Synthesis, characterization and on field evaluation of pesticide loaded sodium alginate nanoparticles. *Carbohydr Polym,* **2014,** *101*(3), 1061–1067.
2. Kodesova, R.; Kocarek, M.; Kodes, V. Pesticide adsorption in relation to soil properties and soil type distribution in regional scale. *J Hazard Mater,* **2011,** *186*(1), 540–550.

## P11 Fragrance from the periphery and beyond: mapping the origins of foreign spices in Chinese materia medica

### Gábor Parti, Chu-Ren Huang

#### Department of Chinese and Bilingual Studies, The Hong Kong Polytechnic University. Hung Hom, Kowloon, Hong Kong SAR, PRC

##### **Correspondence:** Gábor Parti (gabor.parti@polyu.connect.hk)

*Chinese Medicine* 2023, **18(Suppl 1):**P11

Since the legendary “Divine Farmer”, Shennong, and the compendium attributed to him—Shennon Bencao Jing—Chinese people have strived to collect, describe, and categorize medicinal substances. Throughout the centuries, knowledge of these materials was collated into ‘bencaos’, compendiums containing the many plant-based, animal, and mineral products. Among the thousands of materials used in Chinese Medicine however, there is a special group that represent exotic, foreign items. Extra-Chinese herbs and plants were systematically recorded as early as the fourth century in the Nanfang Caomu Zhuang^2^ (Plants of the Southern Regions) [1], and many were consequently incorporated not only into the medicine systems, but into culinary traditions as well. Many of these items have highly aromatic properties and can be categorized today as culinary spices (e.g., ginger, cloves, pepper), and incense (e.g., myrrh, frankincense, sandalwood). In this study, we map the origins of these exotic elements, using, botanical, historical, and geospatial information. Following the steps of the late botanist Prof. Shiu-Ying Hu [2] we also examine the history of when these materials entered China. We present the results using interactive visualizations of the approximate origins and the dates the products have been recorded in Chinese medicinal texts, demonstrating historical and cultural significance of the arrival (and presence) of these materials into the Chinese medicinal domain. Please visit the interactive maps at https://github.com/partigabor/botanical-symposium-2022.

**Figure 1 (abstract P11):**
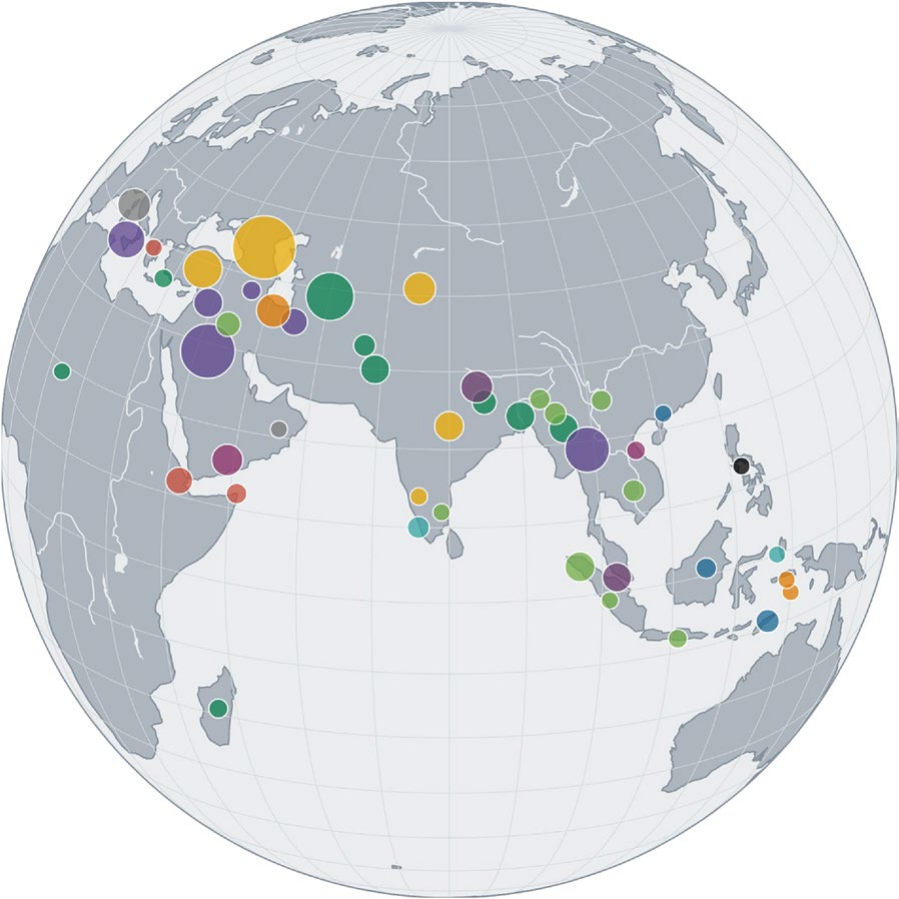
The approximate geographic origins of exotic Chinese medicine from the spice domain

**References**

1. Ji H. *Nan-Fang Ts‘ao-Mu Chuang: A Fourth Century Flora of Southeast Asia*. Chinese University Press; 1979.
2. Hu SY. History of the Introduction of Exotic Elements Into Traditional Chinese Medicine. Journal of the Arnold Arboretum. 1990;71(4):487–526. https://doi.org/10.5962/p.184537

## P12 Kidney-tonifying Chinese herbal medicine *Fructus Ligustri Lucidi* (FLL) promotes myogenesis in C2C12 myoblasts via increasing vitamin D sensitivity

### Christina Chui-Wa Poon^1,2^, Man-Sau Wong^1,2^

#### ^1^Department of Applied Biology and Chemical Technology, Faculty of Science, The Hong Kong Polytechnic University, Hung Hom, Kowloon, Hong Kong SAR, PRC; ^2^Research Centre for Chinese Medicine Innovation, The Hong Kong Polytechnic University, Hung Hom, Kowloon, Hong Kong SAR, PRC

##### **Correspondence:** Christina Chui-Wa Poon (christina.poon@polyu.edu.hk)

*Chinese Medicine* 2023, **18(Suppl 1):**P12

The co-existence of osteoporosis and sarcopenia (also known as osteosarcopenia) in the older adults contributes to their higher risk for falls and fragility fractures due to reduced support from skeletal muscles. *Fructus Ligustri Lucidi* (FLL) is a commonly used Chinese herb for management of musculoskeletal disease as it could effectively preserve healthy energy through nourishing the kidneys and liver based on traditional Chinese medicine (TCM) principle. Oleanolic acid (OA) is one of the major bioactive compounds found in FLL that may account for its anabolic actions in skeletal muscle. In the present study, we hypothesize that FLL and its bioactive compound OA promote myogenesis in C2C12 myoblasts via modulating vitamin D sensitivity. FLL and OA could exert stimulatory effects on myogenesis in which FLL (5 and 10 μg/ml) and OA at 1 nM to 0.1 μM significantly increased the number and area of myotubes (*p* < 0.001) in differentiating C2C12 myoblasts. FLL was found to significantly upregulate the protein expressions of myogenic markers myosin heavy chain (MHC) and myoblast determination protein 1 (MyoD) as well as the expression of vitamin D receptor (VDR) and CYP27B1 in differentiating C2C12 myoblasts. These results suggest that the myogenic effects of FLL might be mediated by an improvement of vitamin D sensitivity. This study provides the evidence for supporting the use of FLL for management of osteosarcopenia.

**Acknowledgements**

This work was supported by the Health and Medical Research Fund of Health Bureau (19200411) and PolyU Start-up Fund for RAPs under the Strategic Hiring Scheme (1-BD4V). We also thank the University Research Facility in Life Sciences at The Hong Kong Polytechnic University for the technical support.

## P13 Local Chinese medicine practitioners’ consensus of traditional Chinese medicine patterns, symptoms and herbal formulas for Hong Kong coronavirus disease 2019 survivors: a modified Delphi study

### Jiayin Ruan^1^, Shucheng Chen^1^, Hoyin Ian Cheng^2^, Wing Fai Yeung^1^

#### ^1^School of Nursing, The Hong Kong Polytechnic University, Hung Hom, Kowloon, Hong Kong Special Administrative Region, China; ^2^Department of Rehabilitation Sciences, The Hong Kong Polytechnic University, Hung Hom, Kowloon, Hong Kong Special Administrative Region, China

##### **Correspondence:** Wing Fai Yeung (jerry-wf.yeung@polyu.edu.hk)

*Chinese Medicine* 2023, **18(Suppl 1):**P13

**Background:** Traditional Chinese Medicine (TCM) has been used to manage COVID-19 survivors. Although the “Novel Coronavirus Pneumonia Recovery Period Guidance for Chinese Medicine Rehabilitation” (the Guideline) was formulated by the Mainland Chinese medicine expert group in April 2022, no consensus is established among Hong Kong Chinese medicine practitioners (CMPs) regarding TCM patterns, symptoms, and herbal formulas in the Guideline. Thus, this study aimed to establish consensus among local CMPs on these items.

**Materials and methods:** A modified Delphi study was conducted between 28 July and 14 September 2022. Each survey gathered feedback using a 5-point Likert scale followed by open-ended questions. Descriptive statistics for quantitative data and thematic analysis for qualitative data were employed. Consensus was defined as ≥ 80% level of agreement with interquartile range (IQR) ≤ 1^[1,2]^.

**Results:** Thirteen local CMPs with clinical experience in managing COVID-19 survivors participated in the three-round survey. A final consensus was reached regarding the applicability of (1) the pattern of *qi deficiency of the lung-spleen* with 6 new suggested symptoms (median = 4; IQR = 0; level of agreement = 92.3%); (2) the pattern of *dual deficiency of qi and yin* with 3 new suggested symptoms (median = 4; IQR = 0.5; level of agreement = 100%); and (3) modified herbal formulas for these two patterns based on panellists’ suggestions. No consensus was achieved for the pattern of *deficiency of qi and yin with phlegm stasis obstructing the collaterals*.

**Conclusion:** The local CMPs-consented adapted Guideline will be more applicable to Hong Kong COVID-19 survivors.

**References**

1. Jorm, A. F. Using the Delphi expert consensus method in mental health research. *Aust N Z J Psychiatry*, **2015,** *49*, 887–897.
2. De Vet, E.; Brug, J.; De Nooijer, J.; Dijkstra, A.; De Vries, N. K. Determinants of forward stage transitions: a Delphi study. *Health Educ Res,* **2005,** *20*, 195–205.

## P14 Effects of traditional Chinese medicine on insomnia-like phenotype and movement deficiency in Zebrafish

### So Chun Pang^1^, Yu Suen Chan^2^, Tian Li^2^, Seto Sai-wang^1^, Daniel Kam-wah Mok^1^, Shuk Han Cheng^2^, Yeung Wing Fai^3^, Zhang Huan^1^

#### ^1^Department of Applied Biology and Chemical Technology, The Hong Kong Polytechnic University, Hung Hom, Kowloon, Hong Kong, China; ^2^Department of Biomedical Sciences, College of Veterinary Medicine and Life Science, City University of Hong Kong, Hong Kong, China; ^3^School of Nursing, The Hong Kong Polytechnic University, Hung Hom, Kowloon, Hong Kong, China

##### **Correspondence:** Yeung Wing Fai (zhanghuan.zhang@polyu.edu.hk); Zhang Huan (jerry-wf.yeung@polyu.edu.hk)

*Chinese Medicine* 2023, **18(Suppl 1):**P14

Insomnia is a common symptom of menopausal women mainly caused by psychological stress^1^. Chronic insomnia might significantly contribute to both mental and physical illnesses, such as depression and hypertension. Traditional Chinese medicines (TCM) have showed to be effective to relief the insomnia-like symptoms^2^. In this study, we have established a zebrafish insomnia model by transition from light to dark. Zebrafish possess orthologs for 86% of 1318 human drug targets which can be used for evaluating the therapeutic beneficial effects for drug candidates^3^. Water extraction of three potentially effective TCM, including Euodiae Fructus, Coptidis Rhizoma and Gardeniae Fructus, have been evaluated by using Zebrafish model. The data have showed that TCM might significantly attenuate the light and dark stimuli-induced insomnia-like phenotype and movement deficiency in zebrafish. The underlying mechanisms will be further investigated by using light and dark stimuli models in Zebrafish. In conclusion, TCM including Euodiae Fructus, Coptidis Rhizoma and Gardeniae Fructus, might help to relief insomnia in menopausal women in the future.

**References**

1. Yuksel, D.; de Zambotti, M.; Sugarbaker, D.; Schulte, T.; Colrain, I. M.; Baker, F. C., Physiological responses to acute psychosocial stress in women with menopausal insomnia. *International Journal of Psychophysiology* **2021,** *164*, 87–94.
2. Wang, L.-X.; Zhao, Q.; Zhang, Y.; Xue, R.; Li, S.; Li, Y.; Yu, J.-J.; Li, J.-C.; Zhang, Y.-Z., Network pharmacology and pharmacological evaluation for deciphering novel indication of Sishen Wan in insomnia treatment. *Phytomedicine* **2023,** *108*, 154500.
3. Rihel, J.; Prober, D. A.; Arvanites, A.; Lam, K.; Zimmerman, S.; Jang, S.; Haggarty, S. J.; Kokel, D.; Rubin, L. L.; Peterson, R. T., Zebrafish behavioral profiling links drugs to biological targets and rest/wake regulation. *Science* **2010,** *327* (5963), 348–351.

## P15 Metabolomics analysis of tetrandrine-treated mouse liver revealed the activity of tetrandine in lipid metabolism

### Hok-Him Tang^1^, Wing-Cheung Chan^1,2^, Chi-on Chan^1^, Ben C. B. Ko^1,2^, Daniel Kam-Wah Mok^1,3^

#### ^1^Department of Applied Biology and Chemical Technology, The Hong Kong Polytechnic University, Hong Kong SAR, China; ^2^State Key Laboratory of Chemical Biology and Drug Discovery, The Hong Kong Polytechnic University, Hong Kong SAR, China; ^3^The Research Centre for Chinese Medicine Innovation, The Hong Kong Polytechnic University, Hong Kong SAR, China

##### **Correspondence:** Ben C. B. Ko (ben.ko@polyu.edu.hk); Daniel Kam-Wah Mok (bcdaniel@polyu.edu.hk)

*Chinese Medicine* 2023, **18(Suppl 1):**P15

Tetrandrine (Tet) is a major secondary metabolite of *Stephania tetrandra S. Moore* (Fen Fang Gi). Tet exhibited potential inhibitory activity towards Ebola virus infection by inhibiting NAADP-induced lysosomal calcium release via the two-pore channels (TPCs) [1]. Our latest study revealed that lysosomal integral membrane protein-2 (LIMP-2), a lysosomal cholesterol transporter, is a direct cellular target of Tet, and a novel regulator of the TPCs. Accordingly, Tet alters cellular cholesterol and fatty acids metabolisms through inhibition of LIMP-2. Moreover, mice treated with Tet for 7 days resulted in the development of hepatic steatosis. To further understand how Tet alters fat metabolism in vivo, livers from Tet-treated mice were subjected to lipidomics analysis using UPLC-Orbitrap-MS. Our results showed that Tet increases the abundance of phospholipids (PL), monoacylglycerides (MAG) and diacylglycerides (DAG), whereas it reduces the abundance of phosphatidylethanolamine (PE) and choline, in a dose-dependent manner. Our study revealed that lipid dysregulation induced by Tet might be preceded by the accumulation of acylglycerides, which are known precursors of many lipids. On the other hand, depletion of choline, which has been known for its role in cholesterol metabolism disorder [2], might play a role in the development of Tet-induced hepatic steatosis. Studies are underway aiming to maximize the therapeutic response of Tet, while minimizing its effect on cholesterol dysregulation.

**References**

1. Sakurai, Y. et al*. Science,* **2015, 347**, 995–998.
2. Govardhan, B; Ravikanth V, V; Sasikala, M. et al*. Journal of Clinical and Experimental Hepatology*, **2019**, *5*, 561–568

## P16 A novel C21 steroidal saponin isolated from Qingyangshen mitigates amyloid-β defects by activating cellular autophagy

### Zi-Ling Tang^1^, Qian-Qian Pang^1^, Xiao-San Li^2^, Yang-Yang Zhang^1^, Jie Zhang^1^, Zuo-Cheng Qiu^1^, Jia-Xu Chen^1^

#### ^1^Guangzhou Key Laboratory of Formula-Pattern of Traditional Chinese Medicine, School of Traditional Chinese Medicine, Jinan University, Guangzhou, China; ^2^School of Pharmacy, Guangdong Medical University, Dongguan, 523808, PR China

##### **Correspondence:** Zuo-Cheng Qiu (zcqiu@jnu.edu.cn); Jia-Xu Chen (chenjiaxu@hotmail.com)

*Chinese Medicine* 2023, **18(Suppl 1):**P16

Qingyangshen (QYS), a traditional Chinese herb medicine, is mainly distributed in the Southwest of China, especially in Yunnan province ^[1]^. Previous chemical study has shown that the main medicinal ingredient of QYS is C21 steroidal saponin which have been reported to have anti-epileptic activity ^[2]^. However, the neuroprotective effect and anti-β-amyloid effect of C21 steroidal saponin are still remain unknown. One C21 steroidal saponin, namely 20-*O*-vanilloyl-kidjoranin (20-O-VK), was extracted from QYS previously ^[3]^. The present study was designed to explore the neuroprotective activity of 20-O-VK on glutamate-induced mouse hippocampal neuronal cells (HT22) death. Moreover, we evaluated its inhibition effect on accumulation of amyloid β plaques(Aβ) of AD model N2a/APP695 cells (N2a cells stably transfected with the human APP gene) and determine if autophagy related proteins are responsible for its regulatory mechanism. Our recent results indicated that 5 and 10 μM of 20-O-VK promote cell proliferative of HT22 cells (p < 0.001). Moreover, it exerts neuroprotective effect on glutamate-induced neuronal death of HT22 cells (p < 0.001). Furthermore, 10 μM of 20-O-VK significantly down-regulated the expression of CTF protein (p < 0.01) and up-related the expression of LC3B II/LC3B I (p < 0.05), without significant effect on full-APP protein (p > 0.05). In conclusion, 20-O-VK treatment can protect HT22 cells from glutamate-induced neuron toxicity, and it also can increase the clearance of excess Aβ accumulation.

**References**

1. Iyaswamy A, Krishnamoorthi SK, Zhang H, et al. Qingyangshen mitigates amyloid-β and Tau aggregate defects involving PPARα-TFEB activation in transgenic mice of Alzheimer’s disease. Phytomedicine. **2021**;91:153648.
2. Li X, Zhang M, Xiang C, Li BC, Li P. Antiepileptic C21 steroids from the roots of Cynanchum otophyllum. J Asian Nat Prod Res. **2015**;17(7):724–732.
3. Li XS, Yang XM, Ding WJ, et al. New C21-steroidal aglycones from the roots of Cynanchum otophyllum and their anticancer activity. Fitoterapia. **2021**;149:104833.

## P17 Anti‑inflammatory effects of *Fructus Ligustri Lucidi* on a human retinal pigment epithelial cell line

### Ka-Ying Wong^1^, Liping Zhou^2,3^, Man-Sau Wong^2,4,5^

#### ^1^Centre for Eye and Vision Research (CEVR), 17W Hong Kong Science Park, Hong Kong; ^2^Department of Applied Biology and Chemical Technology, The Hong Kong Polytechnic University, Hung Hom, Kowloon, Hong Kong; ^3^School of Optometry, The Hong Kong Polytechnic University, Hung Hom, Kowloon, Hong Kong; ^4^State Key Laboratory of Chinese Medicine and Molecular Pharmacology (Incubation), The Hong Kong Polytechnic University Shenzhen Research Institute, Shenzhen 518057, PR China; ^5^Research Center for Chinese Medicine Innovation, The Hong Kong Polytechnic University, Hung Hom, Kowloon, Hong Kong

##### **Correspondence:** Man-Sau Wong (bcmswong@polyu.edu.hk)

*Chinese Medicine* 2023, **18(Suppl 1):**P17

The retinal pigment epithelium (RPE) is a polarized and monolayer of regular polygonal cells arranged at the outer retinal layer [1]. RPE cells is one of the major sources of inflammatory cytokines in the eyes, which activate immune cells in the posterior segment of the eye. Osmolar changes in the eyes could alter RPE functions leading to disturbance of retinal homeostasis and inflammation [2]. *Fructus Ligustri Lucidi* (FLL) is the ripe fruit of *Ligustrum lucidum Ait*, which has been demonstrated to have hepatoprotective effect, anticancer activity, antioxidant activity [3]. The present study further envaulted its function in RPE cells under inflammation condition. Hyperosmotic stress (450 mOsM) was applied to induce inflammation in human retinal pigment epithelial cell line (ARPE-19) by adding sodium chloride to the culture medium before treatment. After 24-h FLL treatment (0.5–20 μg/mL), cell viability was measured by MTS assay and the inflammatory marker mRNA expression levels (IL-6, IL-8 and IL-1β) were measured using Real-time PCR. The results suggested that hyperosmotic stress significantly suppressed the cell viability by 0.75-fold (p < 0.001) and elevated the mRNA expression of IL-6, IL-β by 1.5-fold (p < 0.001) and IL-8 by fourfold (p < 0.001) in ARPE-19 cells compared to the isotonic control group. Also, FLL at 5 and 10 μg/mL significantly reversed the cell viability of APRE-19 cells under hyperosmotic stress by 1.2-fold (p < 0.01). FLL at 10 μg/mL remarkable decreased (p < 0.01) the mRNA expressions of IL-6 by 0.7-fold and IL-8 by twofold compared to the hyperosmotic group. Thus, FLL may have therapeutic potential for managing inflammatory disorders of RPE.

**References**

1. Yang, S., J. Zhou, and D. Li, *Functions and Diseases of the Retinal Pigment Epithelium.* Front Pharmacol, **2021**. 12: p. 727870.
2. Taylor, A.W., S. Hsu, and T.F. Ng, *The Role of Retinal Pigment Epithelial Cells in Regulation of Macrophages/Microglial Cells in Retinal Immunobiology.* Front Immunol, **2021.** 12: p. 724601.
3. Pang, Z., et al., *The advances in research on the pharmacological effects of Fructus Ligustri Lucidi.* Biomed Res Int**, 2015**. 2015: p. 281873.

## P18 The influence of gut microbiota on bone properties and on the bone protective effects of secoisolariciresinol in ovariectomized mice with antibiotic intervention

### Yu-Xin Zhu^1^, Hong-Yu Peng^1^, Man-Sau Wong^1,2,3^, Hui-Hui Xiao^1,2,3^

#### ^1^State Key Laboratory of Chinese Medicine and Molecular Pharmacology (Incubation), The Hong Kong Polytechnic University Shenzhen Research Institute, Shenzhen, 518057, China; ^2^Research Centre for Chinese Medicine Innovation, The Hong Kong Polytechnic University, Hong Kong, China; ^3^Department of Applied Biology and Chemical Technology, The Hong Kong Polytechnic University, Hong Kong, China

##### **Correspondence:** Hui-Hui Xiao (huihui.xiao@polyu.edu.hk)

*Chinese Medicine* 2023, **18(Suppl 1):**P18

Osteoporosis is a metabolic bone disease characterized by low bone mineral density and deterioration of bone microarchitecture. Gut microbiota plays an impart role in maintaining the health of host, especially on bone. Although extensive researches have reported that the digestive system, endocrine system, and immune system all relate to skeletal system via gut microbiota, the regulatory effects of microbiota on bone metabolism were heterogeneous in many studies. This study aims to investigate the influence of gut microbiota on bone properties and on bone protective effects of secoisolariciresinol (SECO) in ovariectomized mice with antibiotics intervention. Four-month old C57 BL/6J mice were employed and subjected to either sham operation (Sham) or bilateral ovariectomy (OVX). The OVX mice were treated with vehicle, E2 (17β estradiol) or SECO. Animals in normal group including Sham, OVX, E2 and SECO mice were given distilled water, while the corresponding antibiotic intervention group were obtained water containing a cocktail of antibiotics. The faecal microbial composition was measured by 16S rRNA sequencing. Ovariectomy significantly decreased bone mineral density (BMD), while SECO reversed this change. Antibiotic intervention led to a significant decrease of BMD in Sham mice, while a significant increase of that in OVX mice. BMD did not change in SECO group with or without antibiotics intervention. The gut microbiota diversities were dramatically suppressed by antibiotics. We concluded that gut microbiota might function differently in sham, OVX or SECO treated mice, supporting microbiota composition altering with the changes of physiology and exogenous substances.

## P19 Effects of piecatannol and resveratrol on high fat diet-induced hyperlipidemia in rats based on quantitative analysis of bile acids

### Siu-Wai Wan^1,2,3^, Chi-On Chan^1^, Huan Zhang^1,2,3^, Yam-Fung Ng^1,2,3^, Shun-Wan Chan^4^, Daniel Kam-Wah Mok^1,2,3^

#### ^1^State Key Laboratory of Chinese Medicine and Molecular Pharmacology (Incubation), Shenzhen, 518057, China; ^2^Research Centre for Chinese Medicine Innovation, The Hong Kong Polytechnic University, Hong Kong, China; ^3^Department of Applied Biology and Chemical Technology, The Hong Kong Polytechnic University, Hong Kong, China; ^4^Faculty of Science and Technology, Technological and Higher Education Institute of Hong Kong, Hong Kong, China

##### **Correspondence:** Daniel Kam-Wah Mok (daniel.mok@polyu.edu.hk)

*Chinese Medicine* 2023, **18(Suppl 1):**P19

This study aims at investigating the effect of piceatannol and resveratrol on bile acid profiles of high fat diet-induced hypercholesterolemic rats. Both piceatannol and resveratrol are naturally available from wine and grapes, and the commercially available supplements. Our previous study suggested that piceatannol is able to prevent hyperlipidemia and there were substantial changes in the serum bile acid profile associated. In this study, rats have been fed with high-fat diet with and without antibiotics for 12 weeks to examine the role of gut microbiota on the effect of piceatannol and resveratrol in preventing hyperlipidemia. Some of the bile acids are the metabolites of the gut microbiota and the bile acids are also affecting the digestion and absorption of lipids in the gut. In order to specifically investigate the gut microbiome via bile acids metabolism, an analytical method quantifying in bile acids has been established using targeted mass spectrometry-based metabolomic platforms. The detail serum bile acid profiles from the in vitro study have been quantified. The LC–MS data have been examined with multivariate statistics to reveal the effect of piceatannol and resveratrol on detail bile acid profile.

Accumulation of conjugated primary bile acids, dominantly taurocholic acids and tauromuricholic acid (*p* < 0.01), and depletion of primary, secondary and conjugated secondary bile acids were found in the antibiotics-treated groups. As for non-antibiotics groups, some secondary bile acids were significantly influenced, such as deoxycholic acid (*p* < 0.01) and 12-ketolithocholic acid (*p* < 0.01), by piceatannol and resveratrol. Moreover, the relative abundance in the total secondary bile acid was elevated, especially taurodeoxycholic acid (*p* < 0.01), in the piceatannol-treated non-antibiotics group. Further confirmation study would be conducted to confirm the role of altered bile acids on the biological effects of piceatannol and resveratrol.

## P20 Immunomodulatory and anti-invasive effects of *Coriolus versicolor*

### Cindy Lai-Hung Yang^1,2,3^, Stanley Chi-Chung Chik^1,2,3^, Allan Sik-Yin Lau^2,3^, Godfrey Chi-Fung Chan^2,3^

#### ^1^BAGI Biosciences, Hong Kong Science Park, Hong Kong Special Administrative Region, China; ^2^Molecular Chinese Medicine Laboratory, Li Ka Shing Faculty of Medicine, The University of Hong Kong, Hong Kong Special Administrative Region, China; ^3^Department of Paediatrics and Adolescent Medicine, The University of Hong Kong, Hong Kong Special Administrative Region, China

##### **Correspondence:** Godfrey Chi-Fung Chan (gcfchan@hku.hk)

*Chinese Medicine* 2023, **18(Suppl 1):**P20

Glioblastoma multiforme (GBM) is one of the most lethal and malignant primary brain tumors. Our previous study demonstrated that tumor necrosis factor (TNF)-α enhanced the invasiveness of T98G glioma cells through matrix metalloprotease (MMP)-3 induction, and such enhancement of cell migration can be inhibited by interferon-gamma^1^. In this study, we hypothesize that the small molecules from *Coriolus versicolor* (SMCV), a premium medicine for enhancing good health and longevity in China for 2000 years, possess immunomodulatory and anti-cancer effects against glioblastoma cells.

The mRNA expression and production of cytokine and MMPs were assessed by quantitative reverse transcription polymerase chain reaction and enzyme-linked immunosorbent assay, respectively. The active compound was identified by nuclear magnetic resonance. The protein expressions of mitogen-activated protein kinases (MAPKs) were measured by Western Blot. The anti-invasive effect of SMCV/active compound was determined using Matrigel® Invasion Chamber.

Results showed that SMCV had strong immunomodulatory effect by suppressing LPS-induced TNF-α production, whereas increasing poly I:C-induced IFN-β level in human primary blood macrophages. SMCV not only possessed indirect anti-cancer effect by suppressing TNF-α-induced MMP-3 production in T98G cells, but also directly reduced the invasion ability of malignant cells (T98G, A549 and MDA-MB-231). Its bioactive compound, 9-KODE methyl ester, inhibited T98G cell invasion and suppressed TNF-α-induced MMP-3 production in T98G cells via the inactivation of p38 MAPK pathway.

Here we provide evidence of the immunomodulatory and anti-cancer effects of SMCV. 9-KODE methyl ester is a potential new drug candidate against the invasion and metastasis of glioblastoma cells.

**Reference**

1. Cheng, S.M.; Xing, B.; Li, J.C.; Cheung, B.K.; Lau, A.S. *International Journal of Cancer*, **2007,** *121*, 1190–1196.

## P21 Anti-metastatic efficacy of natural flavone tricin in mice bearing patient-derived colon tumor spheroids—a case study

### Sin-Guang Chen^1^, Grace Gar-Lee Yue^2^, Julia Kin-Ming Lee^2^, Si Gao^2^, Stephen Chi-Fai Kim^2^, Simon Siu-Man Ng^3^, Monique Simmonds^4^, Pang-Chui Shaw^1,2,5^, Clara Bik-San Lau^1,2^

#### ^1^Li Dak Sum Yip Yio Chin R&D Centre for Chinese Medicine, The Chinese University of Hong Kong, Hong Kong SAR, China; ^2^Institute of Chinese Medicine and State Key Laboratory of Research on Bioactivities and Clinical Applications of Medicinal Plants, The Chinese University of Hong Kong, Hong Kong SAR, China; ^3^Department of Surgery, The Chinese University of Hong Kong; ^4^Royal Botanic Gardens, Kew, London, UK; ^5^School of Life Sciences, The Chinese University of Hong Kong, Hong Kong SAR, China

##### **Correspondence:** Clara Bik-San Lau (claralau@cuhk.edu.hk)

*Chinese Medicine* 2023, **18(Suppl 1):**P21

We previously demonstrated the anti-tumor and anti-metastatic effects of a natural flavone tricin in orthotopic colon tumor-bearing mice, as well as its anti-inflammatory effect in mice with acute colitis [1, 2]. In this study, the effects of tricin on patient-derived colon tumor spheroids were evaluated in vitro and in vivo. Tumor tissues from two colon cancer patients (CC20 AA and CC20 AC) were collected (Table 1), processed, and then cultured in vitro as spheroids for drug screening. The viability of patient-derived spheroids after incubation with tricin was determined using 3D cell viability assay kit. The spheroids were also inoculated subcutaneously in the flank of NOD/SCID mice, followed by administration with tricin (37.5 mg/kg/day, *p.o.*, daily) or chemotherapeutics (5-fluorouracil (16 mg/kg) and oxaliplatin (1.6 mg/kg) *i.p.*, twice a week) for 11–13 weeks. Our in vitro results showed that CC20 AC spheroids were more sensitive towards 48 h tricin treatment than CC20 AA spheroids (Figure 1A, B). Besides, there was no solid xenografts generated in mice inoculated with spheroids, whereas severe metastasis was observed in lungs and livers. Interestingly, for those mice having inoculated with CC20 AA spheroids and received tricin treatment for 11 weeks, the liver metastasis level was significantly lower than that of untreated control mice (Figure 1C). Similarly, those mice with CC20 AC spheroids treated with tricin or chemotherapeutics for 13 weeks resulted in apparent decreases in lung and liver metastasis (Figure 1D, E). In conclusion, this is the first report showing the anti-metastatic efficacy of tricin in mice bearing colon cancer patient-derived spheroids. Further investigation using more colon cancer patients’ samples is crucial for proving the efficacy of tricin in colon cancer treatment.

**References**

1. Li, X.X., et al. *Phytomedicine,* **2021**, *90*, 153625.
2. Yue, G.G.L., et al. *Molecules*, **2020**, *25(16)*, 3730.

**Table 1 (abstract P21):** Characteristics of two patients with colon tissues collected

Sample codes	CC20 AA	CC20 AC
Age	73	68
Metastases at presentation (Y/N)	N	N
Tumor length (cm)	2	2.5
Origin of sample	Ascending colon	Sigmoid
Number of lymph nodes removed	16	14
Tumor/node (TN) staging	T1N1	T4N1
Tumor differentiation (well/moderate/poor)	moderate	moderate
Lymphovascular permeation (Y/N)	Y	Y
Adjuvant chemotherapy (Y/N)	Y	Y
Adjuvant radiotherapy (Y/N)	N	N
Follow up time from surgery (months)	20	17
Recurrence (Y/N)	N	N

**Figure 1 (abstract P21):**
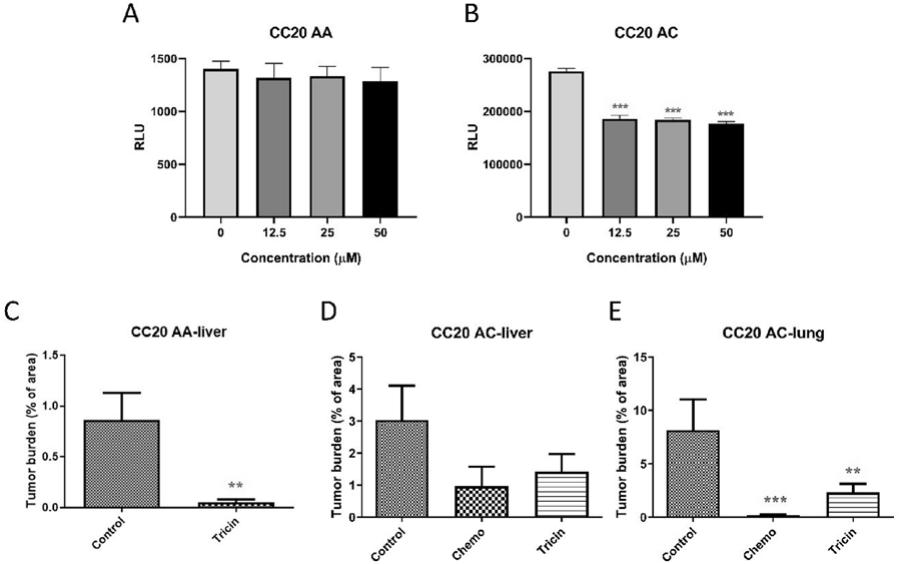
**A**, **B** Cell viability of patient-derived spheroids after 48 h tricin treatment (mean relative light units (RLU) + SD of 2 independent experiments with 3 replicates each). **C**–**E** Effects of tricin or chemotherapeutics on tumor burden in lung or liver in mice bearing patient-derived spheroids (mean + SEM, n = 5–8). **p < 0.01, ***p < 0.001 against untreated control group using one-way ANOVA

## P22 Design, synthesize and characterize the novel quinoline derivatives as the promising anti-Parkinson’s disease candidates by restoring the dysfunction of UCHL1 dependent pathway

### Zhang Huan^1^, Li Chang^2^, Mak Shing-Hung^3^, Lam Kim-Hung^1^, Daniel Kam-Wah Mok^1^, Shun-Wan Chan^3^

#### ^1^Department of Applied Biology and Chemical Technology, The Hong Kong Polytechnic University, Hung Hom, Kowloon, Hong Kong, China; ^2^School of Biomedical Sciences, Faculty of Medicine, The Chinese University of Hong Kong, Shatin, N.T., Hong Kong, China; ^3^Faculty of Science and Technology, Technological and Higher Education Institute of Hong Kong, Hong Kong, China

##### **Correspondence:** Zhang Huan (zhanghuan.zhang@polyu.edu.hk); Shun-Wan Chan (swchan@vtc.edu.hk)

*Chinese Medicine* 2023, **18(Suppl 1):**P22

Parkinson’s disease (PD) is a neurodegenerative disorder characterized by the dysfunctions of movement. The extract causes of PD still remain unknown, around 10% of the cases are considered to be genetically related^1^. Currently, no effective clinical treatment is available for PD by preventing or delaying the pathological progress. The progressive loss of dopaminergic neuron in substantia nigra pars compacta (SNpc) and aggregation of α‑synuclein (α-Syn) protein are the two major pathological features of PD^2^. Autosomal dominant mutation in deubiquitinating enzymes called carboxyl-terminal hydrolase L1 (UCHL1) have been showed to reduce α-syn protein aggregates and neuronal damage^3, 4^. Therefore, development of novel compounds by targeting UCHL1 and α-synuclein might be the strategy for the new generation anti-PD drugs. We have designed and synthesized a series of 8-Hydroxy-2-methylquinoline derivatives and some of them have showed promising protective effects against 6-OHDA-induced neuronal loss in vitro and in vivo. In addition, two quinoline derivatives, Q45 and Q008, promoted the degradation of α-syn, possibly by enhancing α-syn clearance throughUCHL1 dependent pathway. In conclusion, quinoline derivatives, Q45 and Q008, might be the promising multi-functional candidates for the development of new anti-PD drugs.

**References**

1. Feigin, V. L.; Abajobir, A. A.; Abate, K. H.; Abd-Allah, F.; Abdulle, A. M.; Abera, S. F.; Abyu, G. Y.; Ahmed, M. B.; Aichour, A. N.; Aichour, I., Global, regional, and national burden of neurological disorders during 1990–2015: a systematic analysis for the Global Burden of Disease Study 2015. *The Lancet Neurology* **2017,** *16* (11), 877–897.
2. Recchia, A.; Debetto, P.; Negro, A.; Guidolin, D.; Skaper, S. D.; Giusti, P., α‐Synuclein t formatand Parkinson’s disease. *The FASEB Journal* **2004,** *18* (6), 617–626.
3. Ham, S. J.; Lee, D.; Xu, W. J.; Cho, E.; Choi, S.; Min, S.; Park, S.; Chung, J., Loss of UCHL1 rescues the defects related to Parkinson’s disease by suppressing glycolysis. *Science Advances* **2021,** *7* (28), eabg4574.
4. Liu, Y.; Fallon, L.; Lashuel, H. A.; Liu, Z.; Lansbury Jr, P. T., The UCH-L1 gene encodes two opposing enzymatic activities that affect α-synuclein degradation and Parkinson’s disease susceptibility. *Cell* **2002,** *111* (2), 209–218.

## P23 Two common sources of Bai Jiang Cao: Patrinia Herba and Thlaspi Herba—Do they possess the same cytotoxicities in human colon cancer cells?

### Tao Zheng^1^, Dandan Hu^1^, Grace Gar-Lee Yue^1^, Man-Ho Tong^1^, Huihai Yang^1^, Pang-Chiu Shaw^1,2,3^, Clara Bik-San Lau^1,2^

#### ^1^Institute of Chinese Medicine and State Key Laboratory of Research on Bioactivities and Clinical Applications of Medicinal Plants, The Chinese University of Hong Kong, Hong Kong SAR, China; ^2^Li Dak Sum Yip Yio Chin R&D Centre for Chinese Medicine, The Chinese University of Hong Kong; ^3^School of Life Sciences, The Chinese University of Hong Kong, Hong Kong SAR, China

##### **Correspondence:** Clara Bik-San Lau (claralau@cuhk.edu.hk)

*Chinese Medicine* 2023, **18(Suppl 1):**P23

In Mainland China, Bai Jiang Cao (BJC, 敗醬草) can refer to several species, such as *Patrinia scabiosifolia* (Patrinia Herba (PH), Bai Jiang), *Patrinia villosa* (PH, Bai Hua Bai Jiang), *Thlaspi arvense* (Thlaspi Herba (TH), Xi Ming or Su Bai Jiang), or *Sonchus brachyotus* (Ju Mai Cai or Bei Bai Jiang). In Hong Kong, BJC is commonly prescribed alone or in complex prescriptions by Chinese medicine practitioners for cancer treatment, especially colon cancer. However, patients purchasing BJC may be given different species which may then affect the treatment. Therefore, this study aimed to explore which species (PH or TH) could be purchased under the name BJC from herbal stores among the 18 districts in Hong Kong, and comparing the chemistry and cytotoxic activities of these samples.

BJC samples were authenticated morphologically according to literature [1] and their chemical profiles were examined using thin layer chromatography (TLC). Human colon cancer HCT116 cells were employed for evaluating the cytotoxicities of the hot water extracts of these BJC samples using MTT assays [2]. Based on morphology and TLC results, only 5 out of 18 samples were identified as PH, the others were TH (Figure 1). PH extracts were also found to possess cytotoxic activities in HCT116 cells (Table 1), but not TH extracts. In conclusion, our study demonstrated the importance of getting the correct species of BJC in order to obtain the efficacy of such herbal medicine in colon cancer treatment.

**References**

1. He, X., et al*. Am J Chin Med*. **2017**, *45(4)*, 637–666.
2. Yang, H., et al*. Molecules*. **2021**, *26(19)*, 6032.

**Figure 1 (abstract P23):**
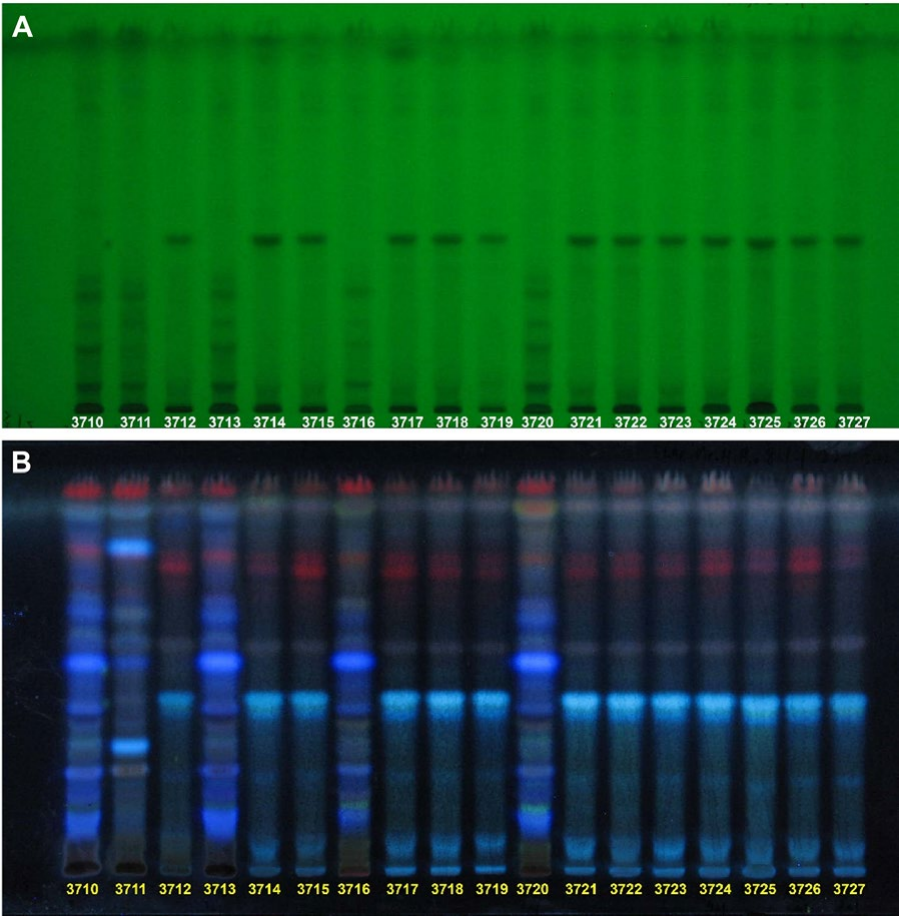
Thin layer chromatograms of 18 BJC samples. Stationary phase: silica gel F254; Mobile phase: EtOAC:MeOH:H_2_O = 8:1:1. View under **A** UV 254 nm, and **B** UV 366 nm after spraying with 10% sulfuric acid–ethanol

**Table 1 (abstract P23):** List of BJC samples and their IC_50_ values in human colon cancer cells

Codes of samples	Districts of Hong Kong	Names on the labels of packaging	IC_50_ in HCT116 cells (μg/ml)
3710	Central and Western	黄花敗醬草 Patriniae Herba	552.0
3711	Wai Chai	No label	772.6
3712	Southern	No label	–
3713	Sham Shui Po	黄花敗醬草 Patriniae Herba	354.5
3714	Kowloon City	No label	–
3715	Wong Tai Sin	No label	–
3716	Kwun Tong	No label	776.4
3717	Kwai Tsing	No label	–
3718	Tsuen Wan	菥蓂(敗醬草) Thlaspi Herba	–
3719	Tuen Mun	菥蓂(敗醬草) Thlaspi Herba	–
3720	Yuen Long	黄花敗醬草 Patriniae Herba	311.0
3721	North	No label	–
3722	Tai Po	菥蓂(敗醬草) Thlaspi Herba	–
3723	Sha Tin	菥蓂(敗醬草) Thlaspi Herba	–
3724	Sai Kung	No label	–
3725	Islands	No label	–
3726	Yau Tsim Mong	菥蓂(敗醬草) Thlaspi Herba	–
3727	Eastern	菥蓂(敗醬草) Thlaspi Herba	–

Note: “–” no cytotoxicity up to 1.6 mg/ml

